# Supplementary material for: Genotype Uniformity, Wild Bird-to-Poultry Transmissions, and Farm-to-Farm Carryover during the Spread of the Highly Pathogenic Avian Influenza H5N8 in the Czech Republic in 2021
Source: Viruses. 2022 Jun 28;14(7):1411. doi: 10.3390/v14071411 (PMC9321741; doi:10.3390/v14071411)
Supplement: Supplementary file 1 [file viruses-1775847-supplementary.pdf › viruses-1775847-supplementary.pdf]

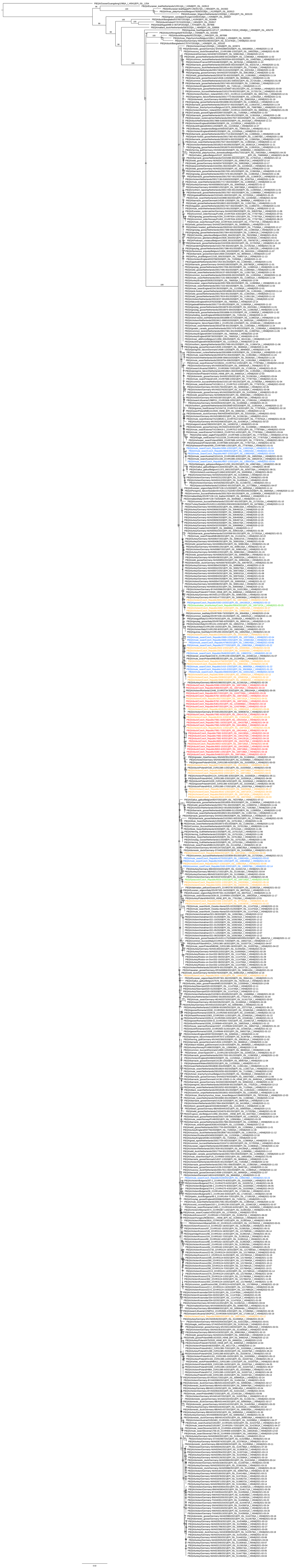

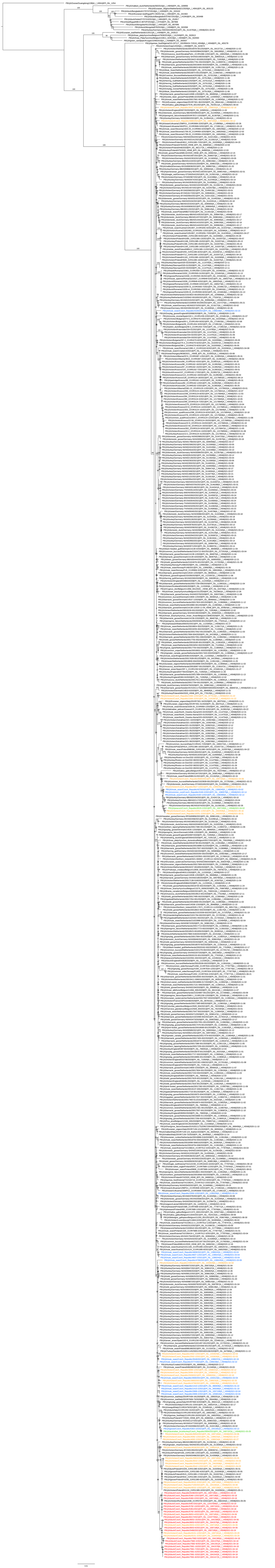

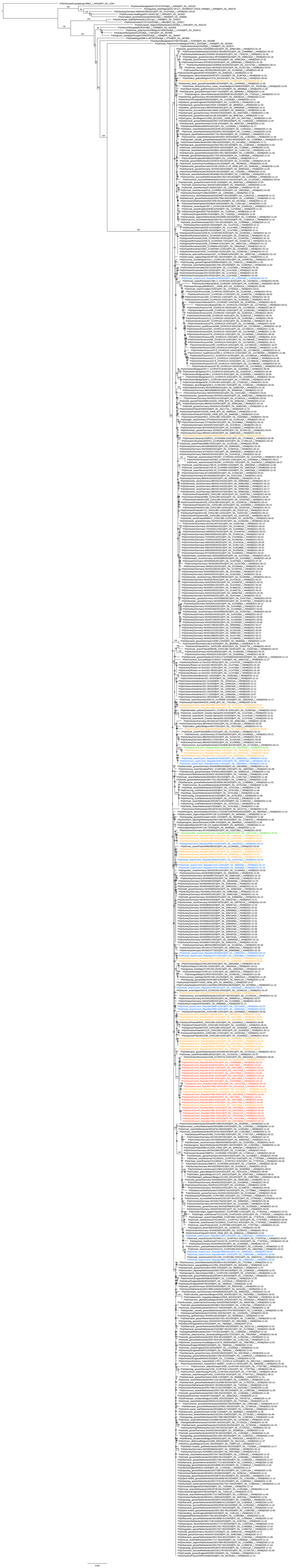

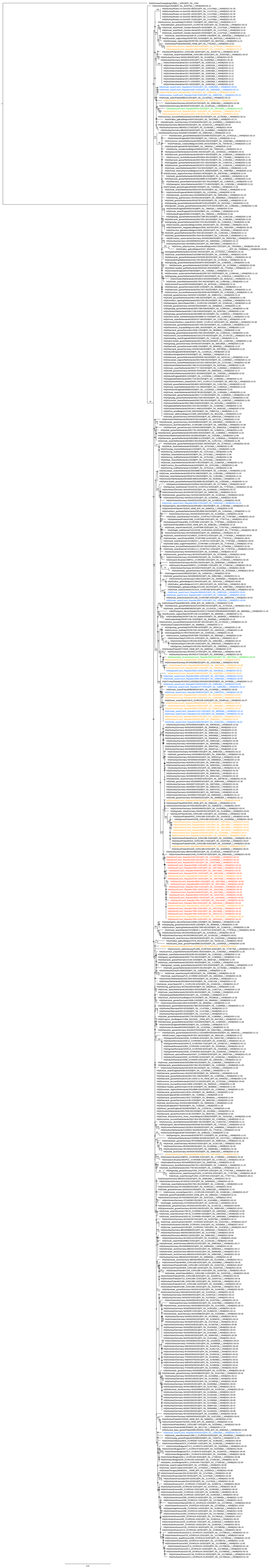

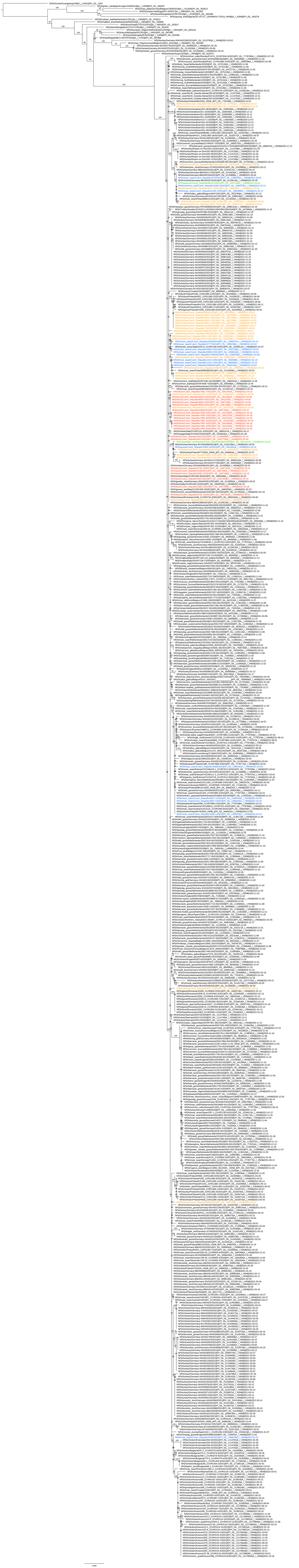
0.009

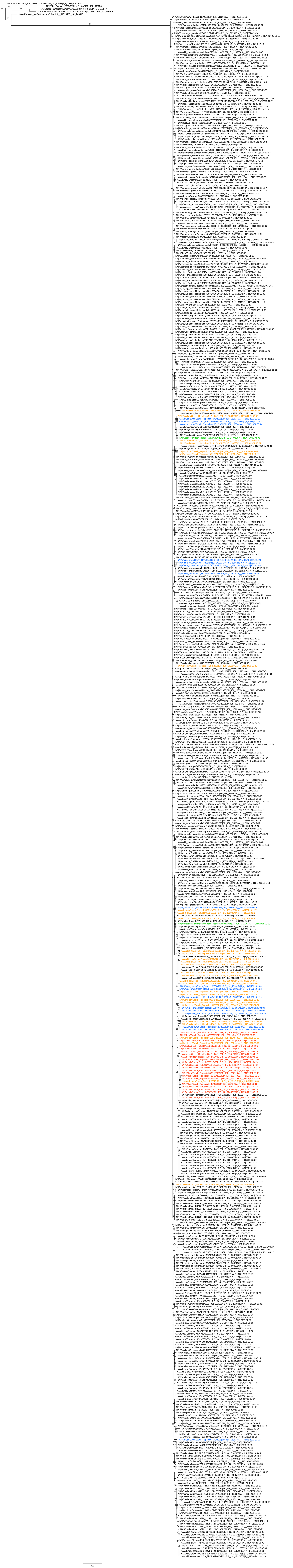

0.02

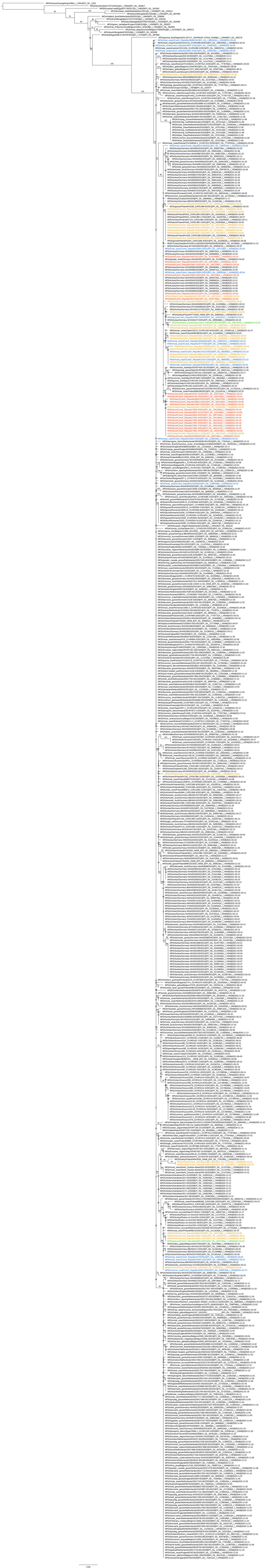

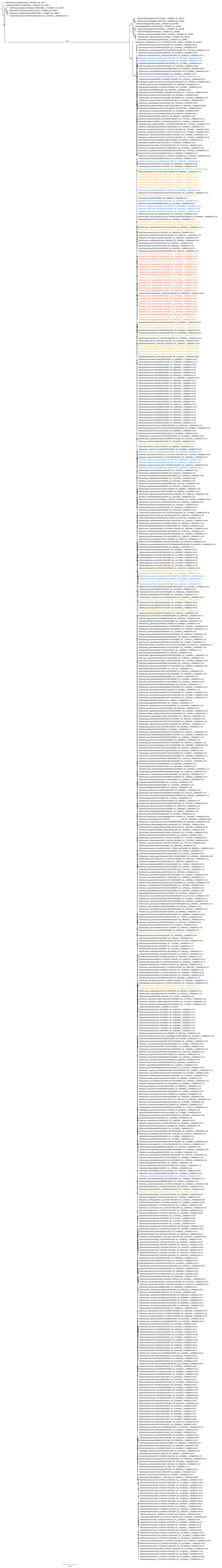

Figure S1. Phylogenetic analysis of CZE/2021 H5N8 genomic segments. The ML tree (Best-fit substitution models according to Bayesian information criterion: PB2: GTR+F+G4; PB1: GTR+F+G4; PA: GTR+F+I+G4; H5: TIM+F+G4; NP: TVM+F+I+G4; N8: UNREST+FO+G4;MP: SYM+G4) was calculated separately for each genomic segment, based on Eurasian H5N8 sequences collected between Nov 2020 and Nov 2021 and stored in the GISAID EpiFlu database PB2 n=584, PB1 n=582, PA=583, H5 n=576, NP=589, N8 n=573, MP n=590 and NS n=590. Colouring: red-commercial, orange-backyard, blue-wild, green-hobby/exotic. For each branch, the bootstrap values (1000 replicates) in percentages are indicated. All trees except N8 were rooted to A/goose/Guangdong/1/1996 H5N1. The N8 tree was rooted to A/mallard/Czech_Republic/14516/2007 H3N8.

Table S1. Pathological signs of the infected birds. The table shows the frequency of pathological signs in absolute values and percentages per bird category represented as a heatmap.

Gross pathology lesions	Chicken n=134	Domestic duck n=30	Wild duck n=5	Goose n=6	Mute swan n=23	Peacock n=1	Common coot n=2
Cyanosis or hyperemia of comb and wattle, head skin or beak	83 (62%)	3 (10%)					
Cardiac or epicardiac hemorrhages, petechiae or inflammation	11 (8%)	4 (13%)			14 (61%)		
Enlarged spleen	119 (89%)	27 (90%)	1 (100%)	5 (83%)	18 (78%)	1 (100%)	
Serosal haemorrhages	33 (25%)	2 (7%)			11 (48%)		
Pancreatic necrosis or haemorrhages	24 (18%)	1 (3%)		6 (100%)	5 (22%)		
Proventricular hemorrhages or erosions	52 (39%)			3 (50%)	7 (30%)	1 (100%)	
Lung hyperemia	66 (49%)	15 (50%)		6 (100%)	13 (57%)	1 (100%)	2 (100%)
Hyperemic trachea	65 (49%)	13 (43%)			1 (4%)		
Leg cyanosis or hyperemia	16 (12%)						
Hepatic necrosis	10 (7%)				9 (39%)		
Hemorrhages in soft tissues of neck					12 (52%)		

%
100
90
80
70
60
50
40
30
20
10

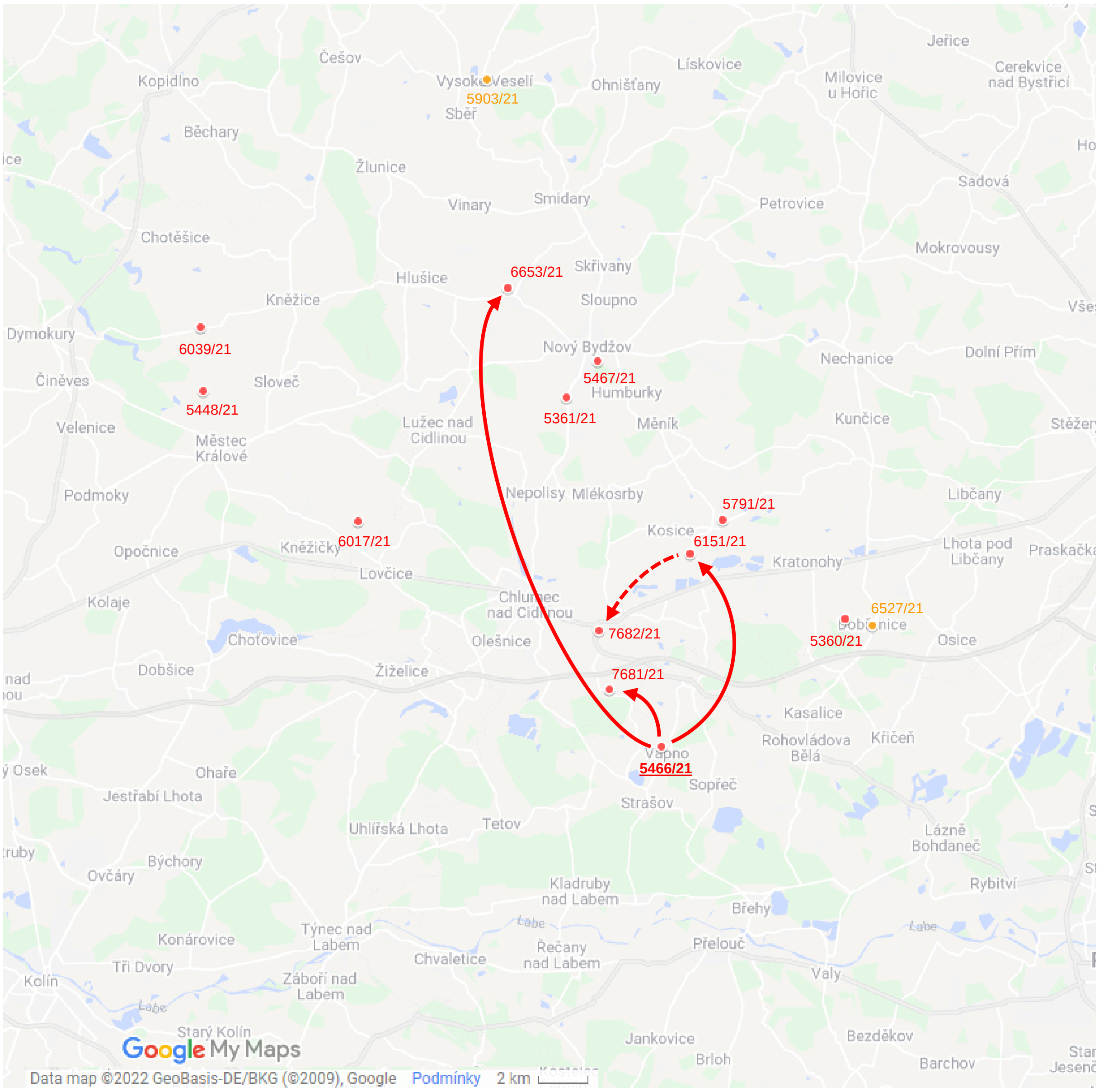

Figure S2. Estimated transmission of H5N8 HPAI between the commercial flocks. The map shows the transmission routes of H5N8 infection inferred for commercial poultry outbreaks belonging to group 3A, as shown in Fig. 3 of the article. Solid arrows designate second-order transmission from the breeding duck flock ID 5466/21. A dashed arrow indicates third-order transmission from commercial hens to breeding ducks.

Geographical distribution of the outbreak localities is provided in the map available on-line: https://www.google.com/maps/d/u/0/viewer?mid=1MNHIwvLUs0sk36Z75OjPGt9h98OqZ&fbclid=IwAR2nXOIkdSAZDUeddMQXcYQH5Y33Yyb2cd5cQ46Ew1dGmjVCFSk_wPhV1e0&ll=50.22443150000001%2C15.479471699999987&z=8
